# Vascular Remodeling in Experimental Hypertension

**DOI:** 10.1100/tsw.2005.122

**Published:** 2005-12-12

**Authors:** Norma R. Risler, Montserrat C. Cruzado, Roberto M. Miatello

**Affiliations:** ^1^ Department of Pathology, School of Medical Sciences, National University of Cuyo, Centro Universitario, (5500) Mendoza, Argentina; ^2^ Department of Morphophysiology, School of Medical Sciences, National University of Cuyo, Centro Universitario, (5500) Mendoza, Argentina

**Keywords:** vascular wall, vascular remodeling, experimental hypertension

## Abstract

The basic hemodynamic abnormality in hypertension is an increased peripheral resistance that is due mainly to a decreased vascular lumen derived from structural changes in the small arteries wall, named (as a whole) vascular remodeling. The vascular wall is an active, flexible, and integrated organ made up of cellular (endothelial cells, smooth muscle cells, adventitia cells, and fibroblasts) and noncellular (extracellular matrix) components, which in a dynamic way change shape or number, or reorganize in response to physiological and pathological stimuli, maintaining the integrity of the vessel wall in physiological conditions or participating in the vascular changes in cardiovascular diseases such as hypertension. Research focused on new signaling pathways and molecules that can participate in the mechanisms of vascular remodeling has provided evidence showing that vascular structure is not only affected by blood pressure, but also by mechanisms that are independent of the increased pressure. This review will provide an overview of the evidence, explaining some of the pathophysiologic mechanisms participating in the development of the vascular remodeling, in experimental models of hypertension, with special reference to the findings in spontaneously hypertensive rats as a model of essential hypertension, and in fructose-fed rats as a model of secondary hypertension, in the context of the metabolic syndrome. The understanding of the mechanisms producing the vascular alterations will allow the development of novel pharmacological tools for vascular protection in hypertensive disease.

> The principal difficulty in your case, remarked Holmes in his didactic fashion, lay in the fact of there being too much evidence. What was vital was overlaid and hidden by what was irrelevant. Of all the facts which were presented to us we had to pick just those which we deemed to be essential, and then piece them together in their order, so as to reconstruct this very remarkable chain of events.
>
> *Sir Arthur Conan Doyle, "The Naval Treaty"*

## INTRODUCTION

Hypertension is a major modifiable risk factor for cardiovascular disease such as ischemic heart disease, cardiac failure, stroke, and end-stage renal disease. Essential hypertension is defined as high blood pressure when a diagnosable known cause is not present, in contrast with secondary hypertension in which known causes such as renovascular disease, renal failure, some adrenal diseases, or other causes are present[1]. Together with the prime sign of an elevated blood pressure, arterial hypertension is characterized by an increase in vascular resistance, cardiac hypertrophy, an increased output of sympathetic nervous system, vascular wall changes, and abnormalities of renal function, among others.

The basic hemodynamic abnormality in hypertension is an increased peripheral resistance that is due mainly to a decreased vascular lumen and derived from functional and structural changes in the small arteries wall. During the first phase of the disease, an altered vascular tone — induced by an increased vasocontraction and decreased vasodilatation — appears, shifting lately to structural shifting, mainly media thickening. At the vascular level, hypertensive changes are associated with humoral and mechanic factors resulting, through signaling events modulation, in abnormal function, growth of cellular components of the vessel wall, and extracellular matrix deposition. These changes, named vascular remodeling, may be adaptive, whereby the hypertrophied vascular wall attempts to normalize arterial wall stress, or maladaptive, where the dysfunctional vessel contributes to increased peripheral resistance and blood pressure elevation[2]. Intensive research has been done and much progress has been made on mechanisms and signaling pathways involved in arterial remodeling in hypertension and in identifying a number of important factors for this process, since the knowledge of these potential therapeutic targets has contributed and will continue to contribute to the development of novel pharmacological tools. Nevertheless, the known factors, and probably the yet unknown, are so many that the mosaic theory of Page[3] for hypertensive disease could be applied to the ethiopathogeny of the vascular remodeling. Because of the complex interrelations of many of these parameters, it can be difficult to evaluate the relative importance of each in an individual manner.

The purpose of this review is to examine the evidence, explaining some of the pathophysiologic mechanisms participating in the development of the vascular remodeling, obtained from experimental models of hypertension, with special reference to the findings in spontaneously hypertensive rats as a model of essential hypertension, and in fructose-fed rats as a model of secondary hypertension, in the context of the metabolic syndrome.

## EXPERIMENTAL HYPERTENSION MODELS

Animal models have been widely used to study etiology and pathogenesis, prevention, therapeutic tools, and risk factors in human diseases[4]. The most widely used rodent models of hypertension have helped scientists to understand the pathogenesis of hypertension and, hence, to develop therapeutic approaches. Basically, animal models encompass primary and secondary hypertension according to etiology. According to Sun et al.[5], primary hypertension includes genetically and environmentally induced hypertension, and secondary hypertension includes pharmacologically induced and renal-induced hypertension. Our group considers that the only models that represent primary hypertension are the genetic models, with variants. All the others, including those obtained by exogenous factors such as stress or diet modifications, constitute models of secondary hypertension.

The genetically induced hypertension models include, among others, the spontaneously hypertensive rats (SHR) originally inbred from the Wistar strain by Okamoto and Aoki, and their WKY inbred normotensive controls. SHR are the most commonly used model for cardiovascular disease and offer the great advantage that their pathophysiological changes are similar to those found in human essential hypertension. Another advantage of the SHR model is that it follows the same progression of hypertension as human hypertension, with prehypertensive, developing, and sustained hypertensive phases, with each phase lasting at least several weeks. Other models are stroke-prone spontaneously hypertensive rats (SHR-SP) and the Dahl salt-sensitive rats, originally derived from Sprague-Dawley stock by Dahl on the basis of developing hypertension with high NaCl diet. The recently developed transgenic models of hypertension open up new possibilities to explore quite specifically the contribution of specific pathophysiological mechanisms[6,7].

A large number of secondary hypertension models has been developed and has demonstrated its usefulness to study different aspects of this disease. Diet-induced hypertension is represented by models of metabolic- or insulin-resistance syndrome, achieved by the chronic administration of carbohydrate-enriched diets. Among them, fructose-fed rats (FFR) provide a model of dietary-induced insulin resistance, which has been used to assess the pathophysiological mechanisms of the metabolic and cardiovascular changes associated with the insulin-resistance syndrome. FFR develop hyperinsulinemia, insulin resistance evidenced by an altered glucose tolerance test, and hypertriglyceridemia; they also develop moderate hypertension and cardiac hypertrophy[8,9].

The DOCA-salt-induced model of hypertension is a typical representative of pharmacologically induced hypertension. Renal-induced hypertension models include two-kidney Goldblatt hypertension (constriction of one renal artery with the contralateral kidney left intact) and one-kidney Goldblatt hypertension (one renal artery constricted and the contralateral kidney removed), imitating human renovascular hypertension. Chronic infusion of the RAS components has also been successful in inducing hypertension. Among them, chronic systemic infusion of angiotensin II (Ang II) at subpressor doses results in slowly progressive increases in blood pressure, providing an experimental model of hypertension that resembles most of the characteristics of essential hypertension found in humans[10]. Chronic intrarenal infusion of Ang II has also been shown to result in preglomerular vascular remodeling and hypertension[11].

Other models that allow the study of specific variables have been also developed, such as the one obtained by blocking nitric oxide (NO) synthesis by chronic administration of L-NAME.

An interesting and exhaustive review on animal models of hypertension has been very recently written by Lerman et al.[12]. A schematic classification of animal models of hypertension and the corresponding human hypertension are shown in Fig. 1.

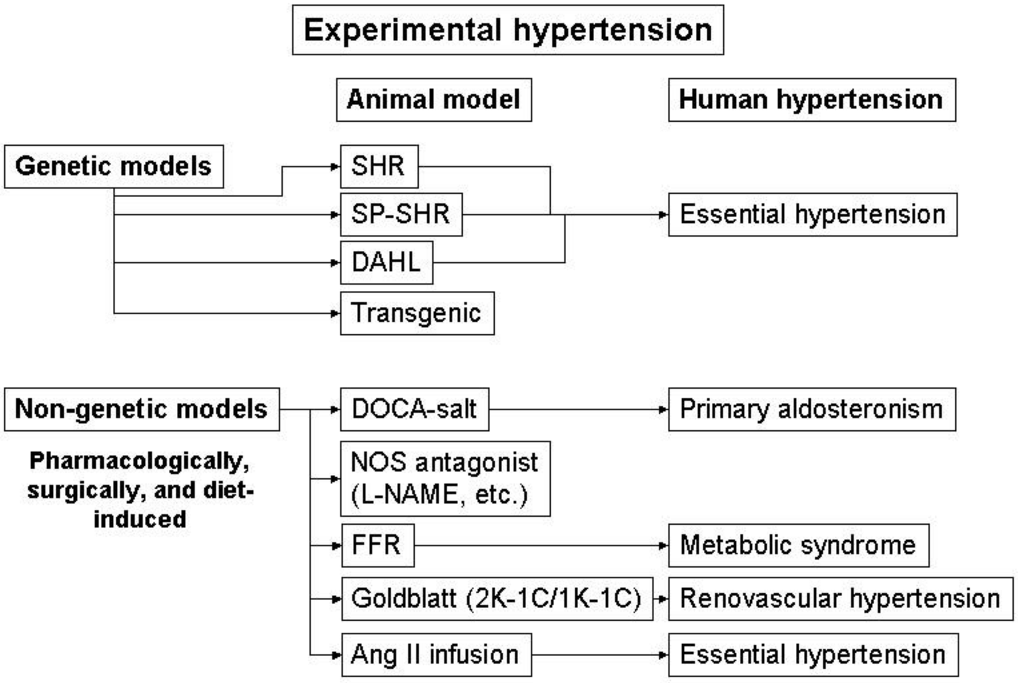

**FIGURE 1.** Animal models of hypertension and the corresponding human hypertension.

## VASCULAR WALL REMODELING

The vascular wall is an active, flexible, and integrated organ made up of cellular (endothelial cells, smooth muscle cells, adventitia cells, and fibroblasts) and noncellular (extracellular matrix) components, which in a dynamic way change shape or number, or reorganize in response to physiological and pathological stimuli, maintaining the integrity of the vessel wall in physiological conditions or participating in the vascular changes in cardiovascular diseases such as hypertension and atherosclerosis[13].

It is beyond question that alteration of the geometric design of resistance arteries is a hallmark of established hypertension and that many of the hemodynamic features associated with essential hypertension can be accounted for by alterations in the structure of the resistance vessels. Altered structure of small arteries may be the first manifestation of target organ damage in hypertensive humans before the appearance of microalbuminuria, thickening of the intima media of carotid arteries, or development of cardiac hypertrophy, among others. It still remains to be established whether remodeling of resistance arteries precedes the development of hypertension or is a consequence of elevated blood pressure.

Let us define what the term vascular remodeling means. In 1989, Baumbach and Heistadt [14] demonstrated that hypertension could be associated with changes in the structure of resistance vessels, such that the vessels had a decreased lumen and increased media to lumen ratio, with no change in media cross-sectional area, and denominated "vascular remodeling" this ability of resistance vessels to change their structure without changing their volume. Since this term was applied lately by cardiologists and vascular biologists to describe any form of change in the cardiovascular system structure, a number of researchers in the field proposed that the term *remodeling* must be used in situations where there is a structurally determined change in lumen diameter, and suggested six patterns of remodeling. Depending on whether the process resulted in a decrease or increase in the diameter, remodeling should be termed inward or outward, respectively. A subclassification into hypertrophic, eutrophic, and hypotrophic remodeling, depending on the increase, no change, or a decrease in the amount of material, respectively, was also agreed[15]. Increase in arterial pressure can induce eutrophic inward remodeling that corresponds to reorganization of cellular and noncellular material of the vascular wall around a reduced lumen, or hypertrophic inward remodeling due to an increase in wall cross-sectional area. Eutrophic inward remodeling of resistance arteries predominates in essential hypertensive patients and in the genetic hypertensive model, SHR, and hypertrophic inward remodeling in secondary hypertension in humans and in rat models of severe hypertension, but both forms of remodeling can coexist in different vascular beds of the same patient or animal model[16]. In arteries that have undergone eutrophic remodeling, the vascular wall has been restructured so that smooth muscle cells (SMC) are aligned more closely and encircle the lumen more tightly without a change in the media volume. Maintenance of media volume may involve a combination of growth and apoptotic processes. It has been proposed that owing to changes in extracellular matrix (ECM) components and corresponding adhesion receptors, interactions between SMC and matrix proteins shift quantitatively, topographically, or both, resulting in a rearrangement of SMC and a restructured vascular wall. In the case of hypertrophic remodeling, growth of the media of a blood vessel results in encroachment of its lumen and may involve increased SMC number, size, or both. SMC growth may be facilitated by several ECM proteins, and the synthetic phenotype of vascular SMC that predominates in hypertension predisposes the vessels to augmented ECM deposition, which is another component of hypertrophic remodeling[17].

In hypertension, blood vessels are regulated by humoral factors, including vasoconstrictor agents such as Ang II, endothelin-1 (ET-1), catecholamines, and vasopressin; vasodilator agents such as endothelium-derived ide NO, endothelium-derived hyperpolarizing factor, and natriuretic peptides; growth factors such as insulin-like growth factor-1 (IGF-1), platelet-derived growth factor (PDGF), epidermal growth factor (EGF), basic fibroblast growth factor (bFGB); cytokines such as transforming growth factor-beta (TGFβ) and interleukines (IL). In addition, there is increasing evidence that reactive oxygen species (ROS) act as intercellular and intracellular signaling molecules and participate in the regulation of vascular tone and structure. Evidence shows that all these factors are related in some way with the pathophysiological mechanisms leading to vascular remodeling in hypertension.

## VASCULAR WALL REMODELING MECHANISMS IN EXPERIMENTAL HYPERTENSION: A FEW OF THE TOO MANY

Studies on vascular structure have been focused mainly on the tunica media with its close-packed components SMC and ECM. However, the adventitia and endothelium are now viewed as key participants in vascular growth and repair[18].

### Adventitia

The adventitia, traditionally considered a structural support for the blood vessel, is emerging as an important player in the pathogenesis of cardiovascular diseases. The role of the adventitia has recently been highlighted in a number of important cardiovascular diseases, including response and repair following direct injury, hypertension vascular remodeling, and atherosclerosis[19]. Quantitative analysis of cell numbers and density has suggested that the adventitial cells are drivers of remodeling and may initiate other changes that supersede alterations in arrangement of SMC and ECM, including impairment of the endothelium-derived NO by its own ROS generation system[20]. Experiments performed *in vitro* and *in vivo* have demonstrated that adventitial cells from rat aortas are an important source of NO generated by the inducible nitric oxide synthase (iNOS), suggesting that adventitial fibroblasts may have an important paracrine role in regulating arterial structure[21]. It has also been shown that mouse aorta adventitial cells, like endothelial cells and SMC, contain a substantial NAD(P)H oxidase superoxide anion generating system, which could contribute to the impairment of the action of endothelium-derived NO[20]. In a very recent review, Yun et al. hypothesize that adventitial dysfunction comprises the dominant source of atherosclerosis by originating many endothelial and smooth muscle abnormalities[19].

### Endothelium

The endothelium, a continuous single-celled layer covering the internal surface of blood vessels, has emerged recently as an organ actively involved in a wide variety of physiological and pathological vascular processes. Endothelial dysfunction has been demonstrated in vessels from hypertensive humans and in many experimental models of hypertension. The endothelium plays a major role in the initiation of vascular remodeling. It serves as a sensor of hemodynamic and humoral variables and a transducer of signals to subjacent vascular SMC. Subsequently, the alterations of SMC growth, migration, differentiation, death, and ECM modifications are responsible for the resulting vascular remodeling[18].

The endothelium releases vasodilating substances such as prostacyclin, NO, and the endothelium-hyperpolarizing factor, as well as vasoconstrictor substances such as thromboxane $A_2$ and endothelin[22]. NO is the key endothelial-derived relaxing factor that plays a pivotal role in vascular tone and reactivity[23]. NO is generated in the endothelial cell through the conversion of the aminoacid L-arginine to nitric oxide and citrulline, by the constitutive catalyzing enzyme $Ca^{2+}$-dependent nitric oxide synthase (eNOS). Another NO synthase isoform (iNOS) present in endothelial cells, macrophages, and vascular SMC is $Ca^{2+}$ independent and inducible by immunological stimuli[24]. NO diffuses from the endothelial cell to the vascular smooth muscle and by increasing cyclic guanosine monophosphate, causes smooth muscle relaxation and vasodilatation.

According to an extensive review of Nava and Lüscher[25] on NO in experimental hypertension, there are animal models of hypertension in which production of NO is increased and some models in which its production is decreased. It has been suggested that two types of experimental hypertension exist in relation to NO. In a normal situation, vasoconstrictor influences are opposed by NO production. In one type of hypertension, an augmented production of vasoconstrictor factors could lead to an increased synthesis of NO

to act as a protective mechanism. In another form of hypertension, with a decrease in NO production, the vasoconstrictor activity in the vascular wall would be unopposed, leading to an increase in blood pressure[26].

SHR results about eNOS activity and NO release are, in some way, controversial[27]. Depending on the stimulus used to increase NO production and other experimental conditions such as rat age or tissue, different biochemical and functional responses of eNOS can be found with reports showing unaltered, diminished[28], or augmented[29] endothelial NO generation. Besides, in SHR, endothelial dysfunction appears to be associated more with enhanced degradation of NO, by superoxide, rather than reduced NO generation[30].

In FFR, a model of secondary hypertension, a decreased endothelial eNOS activity in the endothelial lining of a conduit artery (aorta) and of resistance vessels (mesenteric vascular bed) has been found, as well as in cardiac tissue[31]. These changes were reverted by enalapril-[32] or losartan-induced[33] inhibition of Ang II effect. Since NO has been shown to inhibit vascular SMC proliferation and migration *in vitro* as well as *in vivo*[34,35,36], this finding supports the hypothesis that at an early phase of development of the metabolic syndrome, changes in endothelial function may be related to vascular remodeling and onset or progression of the atherogenic process.

According to Vapaatalo et al.[27], even though there is a lot of evidence supporting a primary and causal association of endothelial dysfunction and NO in experimental hypertension, it seems more plausible that they are causative only in some types of hypertension, having instead a secondary, but still important, participation in the maintenance and further elevation of high blood pressure and the development of organ damages in hypertension.

## Smooth Muscle Cells

Vascular SMC are a major constituent of blood vessel walls and have a function of maintenance of vessel structure. Vascular SMC are dynamic, multifunctional cells that contribute to arterial remodeling through numerous processes, including cell growth (hyperplasia and hypertrophy), apoptosis, elongation of cells, reorganization of cells, and/or altered ECM composition[37].

Normally, fully differentiated SMC of adult arteries are in a quiescent “contractile” state and are not particularly responsive to growth factors or growth regulatory molecules that induce proliferation and cell migration. In response to intimal injury by different stimuli, including high blood pressure states, the cells lose their contractile ability, increase protein secretion, and are more responsive to autocrine and paracrine growth factors. There is a structural reorganization with myofilament loss and cells developing extensive endoplasmic reticulum and large Golgi complexes, and these cells change then to an active “synthetic” phenotype. The growth factors further stimulate SMC hypertrophy and hyperplasia, which leads to continuously elevated vascular resistance[38,39]. On the other hand, vascular SMC migration is an important and well-recognized mechanism related more to other pathologic processes such as intimal hyperplasia and atherosclerosis[39]. Another process found in SMC is apoptosis, defined as gene-regulated cell death. Apoptosis is involved in the fine tuning of media growth. It has been reported in various models of hypertension, being increased in some vascular beds and decreased in others in hypertensive rats[37]. The exact role of apoptosis in arterial remodeling remains unclear and it is unknown whether apoptosis is a growth-associated compensatory and adaptive process or a primary event[17].

In the vascular wall remodeling process, both hypertrophy and hyperplasia of vascular SMC can be found[16]. In eutrophic remodeling, there is no true hypertrophy, since the outer diameter and the lumen are reduced without an increase in the media cross-section, meaning a rearrangement of SMC around a small lumen and hence increased media width and media-lumen ratio, without cell hypertrophy. Instead, hypertrophic remodeling implicates SMC growth (in number or size) leading to an increase in media-cross section and media-lumen ratio.

In experimental models of hypertension, hyperplasia and hypertrophy have been demonstrated to contribute, to varying degrees, to vascular remodeling.

In SHR, vascular SMC show exaggerated growth, possibly associated with genetic abnormalities, such as the expression of complement 3[40] and reflecting intrinsic abnormalities, which apparently are independent of excessive blood pressure. In support of this, we have found an exaggerated DNA synthesis that precedes the increase in blood pressure in cultured SMC derived from mesenteric resistance vessels of 3- to 4-week-old SHR, and stimulated with growth factors, in comparison to cells from age-matched normotensive rats[41]. The different proliferation of vascular SMC from SHR and normotensive WKY rats could be related to a different progression in $G_1$ and $G_2$ phases of the cell cycle[42].

Vascular SMC from SHR are able to produce growth factors and express angiotensinogen, several enzymes as angiotensin-converting enzyme, adhesion molecules, and cytokines[43,44]. On the many humoral and growth factors studied and implicated in the changes of the vascular media in hypertension, Ang II appears to be one of the most important. Ang II, the final effector of the renin-angiotensin system, is generated within the vessel wall and regulates vascular SMC tone and growth, inducing cell hyperplasia and hypertrophy, interacting with other growth factors, or influencing other substances generation, such as ROS or NO[45].

ROS, including superoxide, hydrogen peroxide ($H_2O_2$), and peroxynitrite ($ONOO^-$), are essential signaling molecules, which regulate vascular SMC function[46,47]. In vascular cells, the major source of superoxide is a leukocyte-like, membrane-associated NAD(P)H oxidase[48,49]. Although it is now well established that bioavailabilty of ROS is increased in various models of hypertension[50], it is still uncertain whether vascular oxidative stress is a primary event or a consequence of development of hypertension. Ang II appears to be one of the most important factors regulating NAD(P)H oxidase in the vasculature[50,51]. Ang II stimulates ROS generation derived from NAD(P)H oxidase activity and differentially regulates mitogen-activated protein (MAP) kinases in rat vascular SMC, effects that may contribute to the pleiotropic actions of Ang II in these cells[52]. In SHR, Ang II enhances generation of NAD(P)H oxidase-inducible ROS in mesenteric SMC during the development of hypertension, but not in the prehypertensive phase, this effect being regulated, in part, through insulin-like growth factor-1 receptor (IGF-1R) transactivation[53]. These results, coincident with those of Zalba et al.[54], demonstrating an increased NAD(P)H oxidase-driven generation of superoxide in 30-week-old SHR, but not in 16-week-old SHR, suggest that oxidative stress in vascular SMC from SHR is associated with the elevation of blood pressure, but may not be a primary phenomenon in the pathogenesis of the vascular changes in the genetic model of hypertension. On the contrary, NO generation by SMC from SHR seems to precede the increase in blood pressure. In vascular SMC, the calcium/calmodulin-independent NOS, iNOS, is present and its expression can be induced by cytokines such as interleukin-1β, tumor necrosis factor-α, or bacterial lipopolysaccharides[55,56]. Several attempts have been made in an attempt to see differences in the vascular NO-iNOS system between hypertensive and normotensive rats, but the results are contradictory, with reports showing an increase[57,58], a decrease[59], or no change[60] in NO generated by iNOS or in the enzyme expression. There is a whole body of evidence regarding the countervailing influences between NO and Ang II in the cardiovascular system. It has been suggested that Ang II and NO could be integrated in a homeostatic mechanism aimed at regulating vascular structure and function[61], but conflicting reports also exist on Ang II effects on NO-NOS system. In experiments on cultured aortic SMC obtained from SHR, we have found that unstimulated cells obtained from young SHR, before the development of hypertension, exhibited a significantly smaller iNOS activity and nitrite production than young normotensive control rat cells, and that there were no differences in these variables between hypertensive adult SHR and age-matched normotensive rat aortic cells. Besides, levels of iNOS activity and nitrite accumulation were markedly lower in young prehypertensive SHR than those from mature SHR cells, this difference not being observed between young and adult normotensive rat cells[62]. Additionally, an inhibitory effect of Ang II on iNOS activity was found, without differences between SHR and normotensive rat cells, but greater in adult rats. These results, in accordance with other reports[63,64], allow us to speculate that the early impairment in vascular smooth muscle NO production precedes the development of hypertension in SHR and may play a pathophysiological role in the vascular wall remodeling, independently of the blood pressure elevation in the genetically hypertensive rat model.

In the selected experimental model of secondary hypertension, FFR, changes in vascular SMC also appear as an important mechanism of vascular remodeling. We have found an increased proliferative response to growth factors in primary cultures of aortic and mesenteric SMC isolated from FFR, assessed by an increase in DNA synthesis and an increase in the relative cell number, that was abolished in FFR chronically treated with enalapril or losartan[31,32,33]. It has been suggested that the hyperinsulinemia, a consequence of the insulin-resistant state present in this model and in the human metabolic syndrome, could stimulate vascular SMC proliferation. A proliferative effect of insulin on cultured SMC from the aorta and mesenteric arteries from normal rats has been previously described[65]. The high proliferative status of vascular SMC in this model is accompanied by the already-mentioned decrease in NO synthesis and bioavailability, providing evidence that remodeling of arteries may precede the formation of atherogenic lesions and other forms of target organ damage.

## Extracellular Matrix

Noncellular components of the vascular wall constitute the ECM. In the normal media, SMC are surrounded by ECM molecules and the interaction between SMC and matrix components can significantly influence their ability to respond to growth factors and/or chemoattractants, and can promote the transition of vascular SMC from the contractile to the synthetic phenotype[66]. ECM is the structural and functional support to which cells adhere and on which they grow, migrate, and differentiate. In the normal artery wall, the ECM components are a collection of fibrous proteins such as elastin and collagen, proteoglycans (PGs), and structural-adhesive glycoproteins[67]. The distinct molecules of the vascular matrix are organized into a highly ordered network that is closely associated with the vascular cells producing them. In addition to providing the architectural framework for the artery wall that impacts mechanical support and viscoelasticity, the ECM can regulate the behavior of vascular cells, including their ability to migrate, proliferate, and survive injury[68]. Besides, several enzymes such as matrix metalloproteinases (MMP) and their endogenous inhibitors participate in ECM remodeling and degradation, leading to a precise regulation of ECM composition. Alterations in this regulation are implicated in cardiovascular diseases, including vascular remodeling[69,70].

As a biologically active system, an adequate balance of ECM components is necessary for the normal vascular function[71] and defects in matrix composition or distribution are a factor contributing to the development or maintenance of hypertension. It has been proposed that owing to changes in ECM components and corresponding adhesion receptors, interactions between SMC and matrix proteins shift quantitatively, topographically, or both, resulting in a rearrangement of SMC and a restructured vascular wall[17]. Increased ECM, derived from an increase in some of its components and/or a diminished activity of degrading enzymes, contributes to vessel wall thickening in hypertrophic remodeling or to a reorganization of vessel wall cells in eutrophic remodeling.

Many changes in the vascular wall ECM components have been described in both human and experimental hypertension; described extensively in recently published reviews[17,67,69]. Changes in content or composition of collagen and elastin are more related to the stiffness of both conduit and resistance arteries than to the mechanisms for vascular remodeling[72]. In this review, we will refer to vascular PG changes in experimental hypertension. PGs are important nonfibrous matrix components of the arterial wall that consist of a core protein linked to one or more unusual carbohydrates, the glycosaminoglycans (GAGs), which are composed of repeated disaccharide units and exist in different forms, the sulfated form and the nonsulfated form as hyaluronic acid. These molecules are highly diverse with multiple combinations of core protein and polysaccharide chains. The synthesis of some PGs by various kinds of cells, including vascular SMC[73], is stimulated by growth factors, and PGs function as modulators of growth factors. In SHR, we have studied PG production by SMC obtained from resistance and conduit arteries. In cultured mesenteric SMC from adult SHR, a differential response of several forms of PG synthesis to mitogens, including Ang II, was found, indicating the existence of changes in PG modulation in the resistance vessels of SHR[74]. When basal and stimulated with Ang II or fetal calf serum PG synthesis was tested in cultured mesenteric SMC obtained from young SHR in a period preceding the elevation of blood pressure, a significantly greater amount of extracellular and pericellular sulfated PGs in cells from SHR than in normotensive rat cells was found[41]. These results indicate that changes in PG modulation in media SMC of resistance vessels precede the development of hypertension in SHR and are associated to the early blood pressure elevation in genetically hypertensive rats. Changes in PG synthesis and modulation in vascular SMC from a conduit artery, aorta, in SHR has also been found by us[75], supporting the pathophysiologic role proposed for matrix PGs in the vascular wall alterations associated with hypertension and related vascular diseases such as atherosclerosis.

The natural progressive history of type-2 diabetes mellitus with associated metabolic syndrome and compensatory hyperinsulinemia may result in a remodeling of the ECM prior to the diagnosis of overt type-2 diabetes mellitus. In diabetes, decreased levels of heparan sulfate PGs in the glomerular and endothelial, epithelial, and renal tubular cell basal membrane, affecting the effective filtering function, have been found. Other PGs accumulate in developing atherosclerosis, a highly related disease to the metabolic syndrome[68]. As far as we know, PGs of the vascular wall in experimental models of the metabolic syndrome have not been analyzed, and their study in the future would be of great interest in order to know more about vascular remodeling mechanisms in secondary types of hypertension.

## FINAL RESULT: AN ALTERED VASCULAR STRUCTURE

Changes in all the described mechanisms (and in many others not described in this review) are finally converted into changes in vascular structure. It is beyond question that alteration of the geometric design of resistance arteries is a hallmark of established hypertension and that the structural changes in the resistance arteries produce hemodynamic consequences[76]. Through histological techniques, structural changes in the vascular wall can be studied and the media thickness to lumen diameter ratio measured, which is one of the variables indicating vascular remodeling. Recent studies have demonstrated that the media-lumen ratio of resistance arteries has prognostic significance in relation to cardiovascular events in hypertensive subjects and that some antihypertensive drugs can correct small artery structure[16].

In SHR, there is substantial evidence that, even in young rats when they are still normotensive, their resistance vessels differ from those of normotensive controls. Skov and Mulvaney[77] have observed that the renal afferent arteriole is structurally narrowed in young and adult SHR and that this narrowed afferent arteriole lumen diameter in young rats is a predictor of later development of high blood pressure. It also has been described that the early blockade of the renin-angiotensin system with captopril in SHR exerted protective effects on cardiac and vascular morphology and fibrosis, independently of the reduction of mean arterial blood pressure[78].

In FFR with a secondary moderate hypertension, we have also observed changes in the vascular structure of renal arteries evidenced by a decreased lumen-to-media ratio, that were reverted by the administration of a β blocker, nebivolol[79]. Interestingly, vascular remodeling with a different pattern from that induced by hypertension has been described in preglomerular vessels from kidneys of diabetic-streptozotocin rats without overimposed hypertension[80].

## CONCLUSION

In this review, we have addressed several issues related to the mechanisms leading to vascular remodeling in two models of experimental hypertension, representing essential hypertension or secondary hypertension in humans. Much progress has been made in understanding the mechanisms involved in the changes of the vascular structure and in identifying a number of important substances participating in this process. Because of the complex interrelations between many of these variables, it is difficult to evaluate the relative importance of individual factors, but the past and future research on this kaleidoscope of factors has been and will be the base for the development of novel pharmacological tools for vascular protection in the hypertensive disease.

## BIOSKETCHES

**Norma Risler** received an M.D. in 1971 from the School of Medical Sciences, National University of Cuyo, Argentina and currently is Full Professor of Pathophysiology in the Department of Pathology, School of Medical Sciences, National University of Cuyo, Argentina.

**Montserrat Cruzado** received a Ph.D. in Sciences in 2002 from the School of Chemistry, Biochemistry and Pharmacy, National University of San Luis, Argentina and currently holds the position of Associated Professor of Biochemistry in the Department of Morphophysiology, School of Medical Sciences, National University of Cuyo, Argentina.

**Roberto Miatello** received a Ph.D. in Sciences in 1994 from the School of Biochemistry and Pharmacy, University Juan Agustin Maza, Mendoza, Argentina and currently holds the position of Associated Professor of Pathophysiology, Department of Pathology, School of Medical Sciences, National University of Cuyo, Argentina. Dr. Miatello is a Member of the Career of Scientific Research, National Council of Scientific and Technological Research (CONICET), Argentina.

The authors' past and current research work deals with mechanisms involved in cardiovascular pathology, particularly in diseases such as hypertension and metabolic syndrome. They have published several articles and book chapters on this knowledge area.
